# Genetic variants in the TIRAP gene are associated with increased risk of sepsis-associated acute lung injury

**DOI:** 10.1186/1471-2350-11-168

**Published:** 2010-11-30

**Authors:** Zhenju Song, Chaoyang Tong, Zhan Sun, Yao Shen, Chenling Yao, Jinjun Jiang, Jun Yin, Lei Gao, Yuanlin Song, Chunxue Bai

**Affiliations:** 1 Department of Pulmonary Medicine, Zhongshan Hospital, Fudan University, Shanghai, PR China; 2 Department of Emergency Medicine, Zhongshan Hospital, Fudan University, Shanghai, PR China; 3 Departments of Medicine and Physiology, Cardiovascular Research Institute, University of California, San Francisco, USA

## Abstract

**Background:**

Toll like receptors (TLRs) signaling pathways, including the adaptor protein Mal encoded by the TIRAP gene, play a central role in the development of acute lung injury (ALI). Recently, the *TIRAP *variants have been described association with susceptibility to inflammatory diseases. The aim of this study was to investigate whether genetic variants in *TIRAP *are associated with the development of ALI.

**Methods:**

A case-control collection from Han Chinese of 298 healthy subjects, 278 sepsis-associated ALI and 288 sepsis alone patients were included. Three tag single nucleotide polymorphisms (SNPs) of the TIRAP gene and two additional SNPs that have previously showed association with susceptibility to other inflammatory diseases were genotyped by direct sequencing. The differences of allele, genotype and haplotype frequencies were evaluated between three groups.

**Results:**

The minor allele frequencies of both rs595209 and rs8177375 were significantly increased in ALI patients compared with both healthy subjects (odds ratio (OR) = 1.47, 95% confidence interval (CI):1.15-1.88, P = 0.0027 and OR = 1.97, 95% CI: (1.38-2.80), P = 0.0001, respectively) and sepsis alone patients (OR = 1.44, 95% CI: 1.12-1.85, P = 0.0041 and OR = 1.82, 95% CI: 1.28-2.57, P = 0.00079, respectively). Haplotype consisting of these two associated SNPs strengthened the association with ALI susceptibility. The frequency of haplotype AG (rs595209A, rs8177375G) in the ALI samples was significantly higher than that in the healthy control group (OR = 2.13, 95% CI: 1.46-3.09, P = 0.00006) and the sepsis alone group (OR = 2.24, 95% CI: 1.52-3.29, P = 0.00003). Carriers of the haplotype CA (rs595209C, rs8177375A) had a lower risk for ALI compared with healthy control group (OR = 0.69, 95% CI: 0.54-0.88, P = 0.0003) and sepsis alone group (OR = 0.71, 95% CI: 0.55-0.91, P = 0.0006). These associations remained significant after adjustment for covariates in multiple logistic regression analysis and for multiple comparisons.

**Conclusions:**

These results indicated that genetic variants in the TIRAP gene might be associated with susceptibility to sepsis-associated ALI in Han Chinese population. However, the association needs to be replicated in independent studies.

## Background

Acute lung injury (ALI) and its more severe form, the acute respiratory distress syndrome (ARDS), are syndromes of acute respiratory failure that are characterized by acute pulmonary edema and lung inflammation. ALI remains an important cause of death in the intensive care units (ICU) and few specific therapies are available [1]. Although sepsis, pneumonia, aspiration, trauma, pancreatitis and multiple transfusion are recognized as the most common causes of ALI, only a small fraction of patients with these risk factors develop ALI [2]. Clinical and epidemiological studies have supported the hypothesis that genetic factors might play a part in the development and outcome of ALI [3-10]. Identification of genetic variants may provide new insight into the molecular pathogenesis of ALI and lead to the development of new diagnostic and therapeutic targets [6].

The pathogenetic basis of ALI is incompletely understood. However, emerging evidence has suggested that the severity and outcome of ALI depend significantly on systemic inflammatory response [11]. TLRs recognize a diverse array of pathogens and initiate intracellular signaling via their Toll/interleukin-1 receptor domains, leading to an inflammatory host response [12]. Accumulating evidence has demonstrated that inappropriate activation of TLRs signaling pathways plays an important role in the pathogenesis of ALI [13]. The adaptor protein Mal (TIR domain-containing adaptor protein, TIRAP), encoded by the TIRAP gene, is essential for MyD88-dependent signaling downstream of TLR2 and TLR4. After stimulation of TLR2 or TLR4, Mal triggers a signaling cascade, which culminates in the activation of the nuclear factor-κB (NF-κB) and the subsequent activation of pro-inflammatory genes [14]. Therefore, we considered the *TIRAP *a robust candidate gene for ALI susceptibility.

Two functional SNPs in the TIRAP gene have been found association with inflammatory diseases susceptibility [15-19]. Hawn and coworkers found that the T allele of rs7932766 (C558T), related to lower levels of plasma interlukin-6 (IL-6), was associated with increased susceptibility to meningeal tuberculosis [17]. Recently, another SNP rs8177374 (C/T), which causes a leucine substitution at serine 180 of Mal (S180L), was reported association with susceptibility to pneumococcal disease, bacteremia, malaria, tuberculosis and septic shock [15,16]. S180L leads to an amino acid substitution in which Mal alters TLR2 and TLR4 signaling and thereby protects against excessive or inappropriate inflammation [15,16]. To our knowledge, no studies have addressed the impact of *TIRAP *genetic variants on ALI risk.

Given the importance of exaggerated inflammatory response in the pathogenesis of ALI, and the pivotal role of *TIRAP *in this process, we hypothesized that genetic variants in *TIRAP *might be associated with susceptibility to ALI. Therefore, we performed a prospective study in a Han Chinese sepsis-associated ALI sample set using tag SNP approach to examine this hypothesis.

## Methods

### Study enrollment and design

The present study was reviewed and approved by the Ethics Study Board of Zhongshan Hospital, Fudan University, Shanghai, China (No: 2006-23). Informed written consent was obtained from all subjects or from their legal surrogates before enrollment. Definitions of sepsis and ALI/ARDS were in accordance with the American College of Chest Physicians/Society of Critical Care Medicine Consensus Conference [20] and the American-European Consensus Conference statements (AECC) (Additional file 1: Supplemental Table S1) [21]. All sepsis subjects enrolled had either severe sepsis or septic shock. All patients were selected from the Emergency and Respiratory ICUs at Zhongshan Hospital (Shanghai, China), and were treated according to the Surviving Sepsis Campaign guidelines [22]. Exclusion criteria included age below 18 years, severe chronic respiratory disease, severe chronic liver disease (defined as a Child-Pugh score of > 10), using of high-dose immunosuppressive therapy and AIDS patients. All sepsis patients were screened daily for ALI/ARDS development and those who fulfilled the AECC criteria for ALI/ARDS were considered as ALI cases, which included ALI and ARDS patients; whereas those patients who did not develop ALI/ARDS during hospital stay were considered as sepsis alone patients. Baseline characteristics, source of infection, and Acute Physiology and Chronic Health Evaluation (APACHE) II scores of all patients were obtained during ICU stay. Sex- and age-matched controls were selected from healthy blood donors. Questionnaires including smoking, chronic illness and the history of ALI or sepsis were obtained from all control subjects. Healthy controls were defined as individuals without any recent acute illness, any chronic illness and a history of ALI or sepsis. To reduce the potential confounding from ethnic backgrounds, we only enrolled people with self-reported origin of central Han Chinese, including indigenous people from Zhejiang Province, Jiangsu Province, Anhui Province and Shanghai [23,24].

### SNP selection and genotyping

Tag SNPs were selected based on the data of Han Chinese in Beijing (CHB) from the HapMap project phase II [25]. Three tag SNPs (rs595209, rs3802813 and rs8177375) for the 11.85-kb region encompassing the entire TIRAP gene were identified by Tagger within Haploview using the following tagging criteria: pairwise tagging of the HapMap CHB Population with r^2 ^≥ 0.8 and a minor allele frequency (MAF) ≥ 5%. Additionally, two coding SNPs (rs8177374 predicting Ser180Leu, and rs7932766 predicting Ala186Ala) were also genotyped in this study as they have showed evidence of association with other inflammatory diseases [15-19]. These were not genotyped as part of the HapMap project.

Genomic DNA was extracted from whole blood using FlexiGene DNA Kit (Qiagen Hilden, Germany) following the manufacturer's protocol. Genotyping was performed by direct sequencing at the Chinese National Human Genome Center in Shanghai, China. Using Primer 3 software, we designed two primers to completely incorporate the five SNPs. A 693 bp fragment harboring rs595209 and rs3802813 was amplified using the following primers: forward, 5'-TGGTGAAACCCCGTCTCTAC-3' and reverse, 5'-TGGCACAGCTCGGACACTAT-3'. Another 519 bp fragment harboring rs7932766, rs8177374 and rs8177375 was amplified using the following primers: forward, 5'-GCCAGGCACTGAGCAGTAGT-3' and reverse, 5'-ATGTTCTGAGCCCTTCGTGT-3'. The PCR cycling conditions for both fragments involved a denaturation step at 95°C for 5 min and then 35 cycles of 95°C for 30 s, 57°C for 40 s and 72°C for 45 s, with a final elongation cycle of 72°C for 8 min.

The sequencing reactions were performed using Applied Biosystems BigDye (version 3.1) chemistry (Applied Biosystem, Foster City, CA, USA), and the sequences were resolved using an ABI 3730 Genetic Analyzer. Analyses of the sequence traces were performed using the Staden package and double scored by a second operator [26]. A duplicate were added to each 96-well sample plate for quality assurance and quality control validation of inter-plate discordance, and we placed an extra 10 duplicates into our sample set in order to test for experiment-wide discordance. The data completion rate was 99%.

### Statistical analysis

The demographic variables between different groups were compared by χ^2 ^test for categorical variables and by one-way ANOVA or Student's *t*-test for continuous variables. Genotype distributions were evaluated for departures from Hardy-Weinberg equilibrium by the Haploview v4.1 software [27]. The differences in allele and genotype distributions between groups were examined for statistical significance with χ^2 ^test or with Fisher's exact test when appropriate. P values for genotypic distributions were calculated using the global genotype test. Allele counts in cases and controls were used to calculate the OR and the 95% CI. Multiple logistic regression was used to evaluate if each SNP was independently associated with ALI when adjusted for the potential confounding effects of important clinical variables. When comparing ALI patients to sepsis alone patients, age, gender, body mass index (BMI), history of smoking, diabetes, liver cirrhosis and APACHE II score were included in the multivariate models because of their established association with ALI [28-30]. When comparing ALI patients to healthy controls, age, gender, BMI and history of smoking were included in the multivariate models. A two tailed P-value of < 0.05 was considered statistically significant, whereas a value of corrected P < (0.05/number of tests), was considered significant after Bonferroni correction. The linkage disequilibrium (LD) between SNPs was calculated in terms of r^2 ^values by the Haploview v4.1 software [31]. Two-locus haplotypes were estimated using EM algorithm by Haploview v4.1. The haplotype-specific association was analyzed by Haploview v4.1 and the omnibus test were was analyzed by PLINK [32]. The case/control omnibus test is an H-1 degree of freedom test, where H is the number of different haplotypes. Each haplotype was compared with all other haplotypes as the reference in calculating the OR. The SNP-SNP interaction (epistasis) was also investigated among the SNPs using PLINK [32]. A P-value less than 0.05 was considered statistically significant. The software used for statistical calculations was the SPSS 15.0 (SPSS Inc., Chicago, IL, USA) unless specified.

## Results

### Characteristics of the study population

From February 2006 to August 2009, a total of 278 sepsis-associated ALI (103 ALI, 175 ARDS) and 288 sepsis alone patients were enrolled in this study. An additional population of 298 ethnic-matched healthy blood donors was recruited for comparison. The baseline characteristics of the study population are shown in table 1. The primary source of infection was the lungs, involving 87.1% of the combined sample of sepsis alone and ALI patients. There was no significant difference in age, gender, BMI, diabetes, liver cirrhosis and history of smoking between ALI patients and sepsis alone patients (P > 0.05). Sepsis-associated ALI patients had average a higher APACHE II scores and mortality ratio than sepsis alone patients (P < 0.001), although they were comparable in infection sites.

**Table 1:** Demographic and clinical characteristics of all subjects

	Healthy controls	Acute lung injury patients	Sepsis alone patients	P value
Number	298	278 (175 ARDS)	288	N.A
Age	65.3 ± 11.2	63.1 ± 12.1	64.8 ± 14.4	0.55
Male (%)	175 (58.7%)	166 (59.7%)	176 (61.1%)	0.84
BMI (kg.m^-2^)	21 ± 4	23 ± 8	22 ± 6	0.23
Diabetes	0%	43 (15.5%)	40 (13.9%)	0.60
Liver cirrhosis	0%	12 (4.3%)	9 (3.1%)	0.45
Smoker	125 (41.9%)	120 (43.2%)	117 (40.6%)	0.83
Sepsis Insult				
Lung	N.A	244 (87.8%)	249 (86.5%)	0.64
Abdomen	N.A	15 (5.4%)	15 (5.2%)	0.92
UTI	N.A	5 (1.8%)	7 (2.5%)	0.60
Bloodstream	N.A	10 (3.6%)	11 (3.7%)	0.89
Other	N.A	4 (1.4%)	6 (2.1%)	0.75
APACHE II	N.A	19.5 ± 3.3	15.3 ± 2.6	< 0.001
Mortality (%)	0%	149 (46.4%)	96 (33.7%)	< 0.001

ARDS, acute respiratory distress syndrome; N.A, not available; BMI, body mass index; UTI, urinary tract infection; APACHE, acute physiology and chronic health evaluation.

### Associations of the TIRAP gene SNPs with ALI risk

Of the three tag SNPs, the MAFs of rs595209, rs3802813 and rs8177375 in our sample were 32.1%, 16.3% and 11.6% respectively. The MAFs of the two reported SNPs (rs7932766 and rs8177374) were lower than 5% in our data (1.1% and 2.5%, respectively). The genotype and allele frequencies of the five polymorphisms in all studied subjects are summarized in Additional file 1: Supplemental Table S2. The genotyping success rates ranged from 98% to 99% and did not diverge from Hardy-Weinberg equilibrium (P > 0.05) (Additional file 1: Supplemental Table S2).

**Figure 1:**
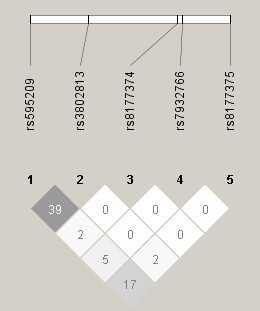
**Linkage disequilibrium plot of five single nucleotide polymorphisms in *TIRAP *genotyped in the current study**. We constructed the plot with the Haploview program [27], and r^2 ^(×100) values were depicted in the diamonds. The color code on the plot follows the r^2 ^scheme: r^2 ^= 0, white; 0 < r^2 ^< 1, shades of grey; r^2 ^= 1, black.

Single locus analysis showed two SNPs (rs595209 and rs8177375) were associated with ALI risk, whereas other three SNPs (rs3802813, rs7932766 and rs8177374) showed no association (Table 2). The alleles of rs595209A and rs8177375G occurred significantly more frequently in the ALI group than in both the healthy control group (OR = 1.47, P = 0.0027; OR = 1.97, P = 0.0001, respectively) and the sepsis alone group (OR = 1.44, P = 0.0041; OR = 1.82, P = 0.00079, respectively), which remained significantly after Bonferroni correction. Moreover, in multivariate analyses after adjustment for covariates, both SNPs were still significantly associated with the development of ALI when compared with healthy control group (OR_adj _= 1.43, P_adj _= 0.0036; OR_adj _= 1.81, P_adj _= 0.00031, respectively) and sepsis alone group (OR_adj _= 1.36, P_adj _= 0.0082; OR_adj _= 1.51, P_adj _= 0.0041, respectively). The genotypes frequencies of rs595209 and rs8177375 in the ALI group were also significantly different from that in the healthy control group (P = 0.0034; P = 0.00047, respectively) and the sepsis alone group (P = 0.0084; P = 0.0026, respectively), the significance remained present in a multivariate analysis controlling for covariates and after Bonferroni correction (Table 2). However, the difference of the allele and genotype frequencies of rs595209 and rs8177375 between subjects with sepsis alone and healthy controls was not statistically significant (P > 0.05) (Additional file 1: Supplemental Table S3). No significant interaction was found between rs595209 and rs8177375 (P > 0.05). The low r^2 ^value between the rs595209 and rs8177375 (r^2 ^= 0.17) indicated the two signals were independent not simply due to LD (Figure 1).

**Table 2:** Association analysis of the five SNPs in *TIRAP *between different groups

SNP	Reference	Acute lung injury patients vs. Healthy controls			Acute lung injury patients vs. Sepsis alone patients		
	allele	OR ( 95% CI)	**P**^**a**^	**P**^**b**^	OR (95%CI)	**P**^**a**^	**P**^**b**^
rs595209	A	1.47 (1.15-1.88)	0.0027	0.0034	1.44 (1.12-1.85)	0.0041	0.0084
		1.43 (1.12-1.82)*	0.0036*	0.0041*	1.36 (1.07-2.02)*	0.0082*	0.0095*
rs3802813	A	1.13 (0.83-1.55)	0.44	0.34	1.03 (0.75-1.41)	0.85	0.61
		1.10 (0.80-1.45)*	0.56*	0.48*	1.01 (0.61-1.24)*	0.91*	0.69*
rs8177374	T	1.43 (0.49-4.14)	0.51	0.59	1.67 (0.54-5.13)	0.85	0.41
		1.28 (0.41-4.34)*	0.63*	0.75*	1.41 (0.51-4.93)*	0.92*	0.55*
rs7932766	T	0.49 (0.21-1.15)	0.095	0.091	0.58 (0.24-1.40)	0.22	0.28
		0.56 (0.28-1.25)*	0.19*	0.23*	0.51 (0.20-1.34)*	0.28*	0.39*
rs8177375	G	1.97 (1.38-2.80)	0.0001	0.00047	1.82 (1.28-2.57)	0.00079	0.0026
		1.81 (1.30-2.69)*	0.00031*	0.00098*	1.51 (1.15-3.04)*	0.0041*	0.0083*

SNP, single nucleotide polymorphism; OR, odds ratio; CI, confidence interval.

P^a^, allelic P value; P^b^, genotypic P value.

* The adjusted P-value and OR in multiple logistic regression after adjustment for clinical variables.

A P-value of < 0.01 (0.05/5) was considered statistically significant after Bonferroni correction. Rs595209 and rs8177375 remained significant after Bonferroni correction.

### Associations of the TIRAP gene haplotypes with ALI risk

We next carried out haplotype analysis on the basis of these two associated SNPs and found that a two-allele model of risk provided the strongest predictor of risk, which was highly significant (P = 0.00006 in ALI group vs control group and P = 0.00003 in ALI group vs sepsis alone group). The two SNPs (rs595209 C/A and rs8177375 A/G) generated three common haplotypes with frequency >5%: CA with frequency of 66.4%, AA with frequency of 22.1% and AG with frequency of 9.8%. A global test showed significant association with omnibus p value of 0.015 in ALI group vs control group and 0.011 in ALI group vs sepsis alone group. In haplotype-specific analysis, the frequency of haplotype AG (rs595209A, rs8177375G) in the ALI samples was significantly higher than that in the healthy control group (OR = 2.13, 95% CI: 1.46-3.09, P = 0.00006) and the sepsis alone group (OR = 2.24, 95% CI: 1.52-3.29, P = 0.00003), suggesting its role as an ALI risk haplotype. After adjustment for covariates, the haplotype AG still associated with increased risk of ALI (Table 3). Another haplotype CA (rs595209C, rs8177375A) appeared protective, as it was found to be more frequent in both healthy control and sepsis alone group than in the ALI group. Carriers of the CA haplotype had a lower risk for ALI compared with healthy control (OR = 0.69, 95% CI: 0.54-0.88, P = 0.0003) and sepsis alone group (OR = 0.71, 95% CI: 0.55-0.91, P = 0.0006). The protective effect of haplotype CA remained significant after adjustment for covariates (Table 3). No association was found between haplotype AA and ALI susceptibility. Similar to the results from individual SNP analysis, the difference in the three haplotypes frequencies between sepsis alone patients and healthy controls was not statistically significant (P > 0.05) (Additional file 1: Supplemental Table S4).

**Table 3:** Association analysis of haplotypes in *TIRAP *between different groups

Haplotypes	Healthy control	Acute lung injury	Sepsis alone	**P**^**a**^	**OR**^**a **^**(95% CI)**	**P**^**b**^	**OR**^**b **^**(95% CI)**
rs595209, rs8177375	(Frequency)	(Frequency)	(Frequency)				
Global test				0.015		0.011	
				0.016*		0.014*	
CA	0.678	0.602	0.686	0.0003	0.69 (0.54-0.88)	0.0006	0.71 (0.55-0.91)
				0.00057*	0.75 (0.60-0.91)*	0.0009*	0.72 (0.54-0.93)*
AA	0.224	0.228	0.218	0.65	1.07 (0.81-1.41)	0.78	1.04 (0.79-1.38)
				0.52*	1.12 (0.87-2.17)*	0.42*	1.21 (0.54-2.06)*
AG	0.076	0.157	0.080	0.00006	2.13 (1.46-3.09)	0.00003	2.24 (1.52-3.29)
				0.0002*	1.78 (1.35-2.92)*	0.0004*	2.09 (1.41-3.01)*

OR, odds ratio; CI, confidence interval.

P^a ^and OR^a ^, acute lung injury vs healthy control; P^b ^and OR^b ^, acute lung injury vs sepsis alone.

* The adjusted P-value and OR in multiple logistic regression after adjustment for clinical variables.

A P-value of < 0.025 (0.05/2) was considered statistically significant after Bonferroni correction. Haplotypes CA and AG remained significant after Bonferroni correction.

## Discussion

TLRs and their signaling pathways play a central role in the initiation of host immune response [12]. Previous studies have described that excessive activation of host immune response contributed to the overproduction of proinflammatory cytokines and the development of ALI or sepsis [13]. Recently, several variants in the TLR signaling pathways genes have been reported to influence the production of inflammatory cytokines and associate with susceptibility to sepsis [15,17,33,34]. However, no studies have addressed the impact of genetic variants in TLR signaling pathways on susceptibility to or outcome of ALI. To our knowledge, this was the first study to investigate the association between the genetic variants of *TIRAP *and the risk ALI. We found that the A allele of rs595209 and the G allele of rs8177375 were significantly associated with the increased risk of ALI. Consistent with the single SNPs analysis, two haplotypes constructed by rs595209 and rs8177375 were also associated with the risk of ALI. These associations remained significant after correction for multiple testing. However, the distribution of *TIPAP *polymorphisms and haplotypes were not significantly different between healthy control and sepsis alone patients. Taken together, our results provided strong evidence that *TIPAP *variants are associated the development of sepsis-associated ALI.

TIRAP/Mal, a bridging adapter in the TLRs signaling pathway, plays a significant role in the pathophysiology of ALI and contributes to morbidity and mortality in both animal models and humans [35]. TIRAP-deficient mice was resistant to the toxic effects of lipopolysaccharide, with defective induction of TNF-α, IL-6 and IL-12, delayed activation of NF-κB and MAP kinases. Protein leak and neutrophil recruitment in the lung were also abrogated in the TIRAP-deficient mice [36,37]. Although rs8177374 and rs7932766 that influence inflammatory cytokine production were found association with various inflammatory diseases, we did not find any association between these two SNPs and ALI susceptibility in the current study. Previous studies showed that the minor allele frequencies of both rs8177374 and rs7932766 were high in West-Eurasian but rare in Asian populations [15,38]. The allele and genotype frequencies of these two SNPs in our study subjects were consistent with that in Asian populations from the previous studies [15-17]. It was important to note that, given the low minor allele frequencies, our power to detect an association for these two polymorphisms was limited. Assuming the prevalence of 0.01 and the OR of 1.5 and using a significance level of 0.05, our study had only 11.9% and 11.8% power to detect association with rs8177374 (MAF of 1.0%) in 278 acute lung injury patients and 288 sepsis alone patients vs. 298 control respectively, and had 25.0% and 24.7% power to detect association with rs7932766 (MAF of 2.9%) in 278 acute lung injury patients and 288 sepsis alone patients vs. 298 control respectively. A large-scale case-control study in Han Chinese population should be performed to evaluate the association between these two polymorphisms and ALI susceptibility.

When compared with the genotypes of other populations from HapMap, we found the rs8177375A allele frequency in our healthy controls and those of Asian descent (CHB), Europeans descent (CEU) and African descent (YRI) from Hapmap does not vary significantly. The minor rs595209 A allele associated with sepsis related ALI has the frequency of 29.6% in the current 298 healthy controls, similar to the Hapmap CHB data (34.3%). However, the A allele of rs595209 is the major allele with a frequency of 93.2% in YRI and 83.2% in CEU from Hapmap data. It remains to be determined whether these differences between ethnic groups influence susceptibility to sepsis related ALI. Investigation in other population is also expected to determine whether the findings is Chinese population specific.

Rs595209 and rs8177375 were reported for the first time to be associated with the susceptibility of ALI. These two SNPs were both located in the non-coding region of TIRAP. SNP rs595209 is located in the intron region of *TIRAP*. Although rs595209 is at the neighboring region of the nonsynonymous SNPs rs8177374 and rs7932766 in the DNA sequence, these SNPs are not in high LD with each other (Figure 1). SNP rs8177375 was located in the 3' untranslated region (UTR) of the transcript NM_148910 and in the intron region of NM_001039661. It is well known that 3' UTRs are regulatory elements which can control protein expression, primarily through effects on mRNA stability and also through transcript translatability [39]. Therefore, it is highly probable that rs8177375 alter the structure of the 3' UTRs, consequently influence the expression of NM_148910. However, given that these were tag SNPs, it is more likely that rs595209 and rs8177375 are tagging other common or rare variants of the TIRAP gene associated with ALI. Another possibility is that the association might be due to LD with variants from nearby genes. Exhaustive resequencing is required to find or rule out the possibility an as-yet-unidentified causal SNP in LD with rs595209 and rs8177375. And further functional studies are needed to investigate whether the variants have an effect on *TIRAP *mRNA stability and translatability.

This study has a number of strengths. First, a sepsis without ALI group was used for comparison to exclude the possibility of a false association with sepsis. Second, to minimize racial admixture, we focused on central Han Chinese patients, which could be regarded as one single homogenous population [23,24]. Third, to reduce the heterogeneous etiologies for ALI, the present study only included patients whose primary etiology for ALI was sepsis. Of note, a major limitation of our study is the lack of independent samples to validate the associations. Additionally, we did not resequence the gene and instead used publicly available SNP databases. Thus, some variants could have been missed due to incompleteness of these databases.

## Conclusions

In conclusion, we reported for the first time that two tag SNPs in the TIRAP gene contribute to increased risk of sepsis-associated ALI in Han Chinese population. However, as this was the first study to analyze the genetic variants in *TIRAP *and ALI risk, future studies are needed to validate the associations in other populations and exhaustively resequence of the TIRAP gene region.

## Abbreviations

TLRs: toll like receptors; ALI: acute lung injury; SNP: single nucleotide polymorphism; OR: odds ratio; CI: confidence interval; ARDS: acute respiratory distress syndrome; ICU: intensive care unit; TIRAP: TIR domain-containing adaptor protein; NF-κB: nuclear factor-κB; IL-6: interlukin-6; AECC: American-European consensus conference statements; APACHE: acute physiology and chronic health evaluation; MAF: minor allele frequency; BMI: body mass index; LD: linkage disequilibrium; UTR: untranslated region.

## Competing interests

The authors declare that they have no competing interests.

## Authors' contributions

CXB and CYT conceptualized and supervised the study. ZJS designed the study, carried out the statistical analysis and wrote the manuscript. ZS, YS, CLY, JJJ and JY were involved with in the recruitment of the patients and controls. YLS helped in preparing the manuscript. LG helped with conducting the experiments.

All authors read and approved the final manuscript.

## Pre-publication history

The pre-publication history for this paper can be accessed here:

http://www.biomedcentral.com/1471-2350/11/168/prepub

## Supplementary Material

Additional file 1 **Supplemental Table S1. The definitions of sepsis, severe sepsis, septic shock and ALI/ARDS**. The definitions for sepsis, severe sepsis, septic shock and ALI/ARDS by the American College of Chest Physicians/Society of Critical Care Medicine Consensus and the American-European consensus conference statements. Supplemental Table S2. Allele and genotype frequencies of the TIRAP gene SNPs in all the subjects. Allele and genotype frequencies of rs595209, rs3802813, rs8177375, rs8177374 and rs7932766 in the healthy controls, ALI patients and sepsis alone patients. Supplemental Table S3. Association analysis of the five SNPs in *TIRAP *between sepsis alone and healthy control groups. Association analysis of rs595209, rs3802813, rs8177375, rs8177374 and rs7932766 between healthy controls and sepsis alone patients. Supplemental Table S4. Association analysis of haplotypes in *TIRAP *between sepsis alone and healthy control groups. Association analysis of three haplotypes (CA, AA and AG) between healthy controls and sepsis alone patients. Click here for file
