# Supplementary material for: Genetic variants in the TIRAP gene are associated with increased risk of sepsis-associated acute lung injury
Source: BMC Med Genet. 2010 Nov 30;11:168. doi: 10.1186/1471-2350-11-168 (PMC3001691; doi:10.1186/1471-2350-11-168)
Supplement: Additional file 1 — Supplemental Table S1. The definitions of sepsis, severe sepsis, septic shock and ALI/ARDS. The definitions for sepsis, severe sepsis, septic shock and ALI/ARDS by the American College of Chest Physicians/Society of Critical Care Medicine Consensus and the American-European consensus conference statements. Supplemental Table S2. Allele and genotype frequencies of the TIRAP gene SNPs in all the subjects. Allele and genotype frequencies of rs595209, rs3802813, rs8177375, rs8177374 and rs7932766 in the healthy controls, ALI patients and sepsis alone patients. Supplemental Table S3. Association analysis of the five SNPs in TIRAP between sepsis alone and healthy control groups. Association analysis of rs595209, rs3802813, rs8177375, rs8177374 and rs7932766 between healthy controls and sepsis alone patients. Supplemental Table S4. Association analysis of haplotypes in TIRAP between sepsis alone and healthy control groups. Association analysis of three haplotypes (CA, AA and AG) between healthy controls and sepsis alone patients. [file 1471-2350-11-168-S1.DOC]

**Supplemental Table S1. The definitions of sepsis, severe sepsis, septic shock and ALI/ARDS**

|  | Definition |
| --- | --- |
| Sepsis | Known or suspected source of systemic infection plus at least two of the following: a) temperature > 38°C or < 36°C; b) heart rate > 90 beats/min; c) respiratory rate > 20 breaths/min or PaCO_2_ < 32 mmHg; d) WBC count > 12,000/mm^3^, < 4000/mm^3^, or > 10% bandemia. |
| Severe sepsis | Sepsis associated with organ dysfunction, hypoperfusion, or hypotension. Hypoperfusion and perfusion abnormalities may include, but are not limited to lactic acidosis, oliguria, or an acute alteration in mental status. Organ dysfunction parameters: a) arterial hypoxemia (PaO_2_/FiO_2_ < 300); b) acute oliguria (urine output <0.5 ml kg^−1^ h^−1^ or 45 mM/l for at least 2 h); c) creatinine increase ≥0.5 mg/dl; d) coagulation abnormalities (international normalized ratio >1.5 or activated partial thromboplastin time >60 s); e) ileus (absent bowel sounds); f) thrombocytopenia (platelet count <100,000/μl); g) hyperbilirubinemia (plasma total bilirubin > 4 mg/dl or 70 mmol/l). |
| Septic shock | Fulfill requirements for sepsis plus one of the following: a) Systolic arterial pressure below 90 mmHg, mean arterial pressure lower than 60, or a reduction in systolic blood pressure of more than 40 mmHg from baseline, despite adequate volume resuscitation, in the absence of other cause of hypotension.; b) need for vasopressors to maintain systolic arterial pressure ≥ 90 mmHg, mean arterial pressure ≥ 60 mmHg or within 40 mmHg of baseline. |
| ALI/ARDS | A a) acute onset of respiratory symptoms; b) chest radiograph with bilateral infiltrates；c) pulmonary artery wedge pressure (PAWP) of less than 18 mmHg or no evidence of left heart failure; d) ALI: PaO2/FiO2 ratio < 300 mmHg; ARDS: PaO2/FiO2 ratio < 200 mmHg. |

ALI, acute lung injury; ARDS, acute respiratory distress syndrome

**Supplemental Table S**2. Allele and genotype frequencies of the TIRAP gene SNPs in all the subjects

| SNP | HWE *P* value | Healthy controls | Acute lung injury patients | Sepsis alone patients |
| --- | --- | --- | --- | --- |
| rs595209 | 0.428 |  |  |  |
| CC |  | 145 (49.3%) | 115 (41.8%) | 140 (49.5%) |
| AC |  | 124 (42.2%) | 111(40.4%) | 117 (41.3%) |
| AA |  | 25 (8.5%) | 49 (17.8%) | 26 (9.2%) |
| C |  | 414 (70.4%) | 341 (62%) | 397 (70.1%) |
| A |  | 174 (29.6%) | 209 (38%) | 169 (29.9%) |
| rs3802813 | 1.0 |  |  |  |
| GG |  | 210 (71.7%) | 182 (67.4%) | 196 (69.3%) |
| AG |  | 75 (25.6%) | 83 (30.7%) | 79 (27.9%) |
| AA |  | 8 (2.7%) | 5 (1.9%) | 8 (2.8%) |
| G |  | 495 (84.5%) | 447 (82.8%) | 471 (83.2%) |
| A |  | 91 (15.5%) | 93 (17.2%) | 95 (16.8%) |
| rs8177374 | 1.0 |  |  |  |
| CC |  | 285 (97.9%) | 265 (97.1%) | 278 (98.2%) |
| CT |  | 6 (2.1%) | 8 (2.9%) | 5 (1.8%) |
| C |  | 576 (99.0%) | 538 (98.5%) | 561 (99.1%) |
| T |  | 6 (1.0%) | 8 (1.5%) | 5 (0.9%) |
| rs7932766 | 1.0 |  |  |  |
| CC |  | 275 (94.2%) | 267 (97.1%) | 270 (95.1%) |
| CT |  | 17 (5.8%) | 8 (2.9%) | 14 (4.9%) |
| C |  | 567 (97.1%) | 542 (98.5%) | 554 (97.5%) |
| T |  | 17 (2.9%) | 8 (1.5%) | 14 (2.5%) |
| rs8177375 | 0.933 |  |  |  |
| AA |  | 241 (81.7%) | 188 (68.4%) | 230 (81.0%) |
| AG |  | 52 (17.6%) | 80 (29.1%) | 50 (17.6%) |
| GG |  | 2 (0.7%) | 7 (2.5%) | 4 (1.4%) |
| A |  | 534 (90.5%) | 456 (82.9%) | 510 (89.8%) |
| G |  | 56 (9.5%) | 94 (17.1%) | 58 (10.2%) |

SNP, single nucleotide polymorphism; HWE, Hardy-Weinberg equilibrium

**Supplemental Table S3. Association analysis of the five SNPs in *TIRAP*** between sepsis alone and healthy control groups

| SNP | Reference allele | P^a^ | Padj^a^ | P^b^ | Padj^b^ | OR ( 95% CI) | ORadj ( 95% CI) |
| --- | --- | --- | --- | --- | --- | --- | --- |
| rs595209 | A | 0.92 | 0.84 | 0.95 | 0.88 | 1.01 (0.79-1.30) | 1.08 (0.75-1.35) |
| rs3802813 | A | 0.56 | 0.67 | 0.81 | 0.85 | 1.10 (0.80-1.50) | 1.05 (0.86-1.45) |
| rs8177374 | T | 0.80 | 0.76 | 0.80 | 0.74 | 0.86 (0.26-2.82) | 0.84 (0.22-2.88) |
| rs7932766 | T | 0.64 | 0.69 | 0.64 | 0.70 | 0.84 (0.41-1.73) | 0.89 (0.46-1.82) |
| rs8177375 | G | 0.68 | 0.59 | 0.69 | 0.56 | 1.08 (0.74-1.60) | 1.14 (0.84-1.78) |

SNP, single nucleotide polymorphism; OR, odds ratio; CI, confidence interval.

P^a^, allelic P value; P^b^, genotypic P value.

* The adjusted P-value and OR in multivariate analyses after adjustment for age, gender, body mass index and history of smoking.

**Supplemental Table S4. Association analysis of haplotypes in *TIRAP* between** sepsis alone and healthy control groups

| Haplotypes | Healthy control | Sepsis alone | P | OR (95% CI) |
| --- | --- | --- | --- | --- |
| rs595209, rs8177375 | (Frequency) | (Frequency) |  |  |
| Global test |  |  | 0.98 |  |
|  |  |  | 0.96* |  |
| CA | 0.678 | 0.686 | 0.85 | 0.98 (0.76-1.25) |
|  |  |  | 0.77* | 0.95 (0.67-1.21)* |
| AA | 0.224 | 0.218 | 0.87 | 1.02 (0.78-1.35) |
|  |  |  | 0.81* | 1.09 (0.67-1.57)* |
| AG | 0.076 | 0.080 | 0.82 | 0.95 (0.62-1.46) |
|  |  |  | 0.91* | 0.98 (0.85-1.32)* |

OR, odds ratio; CI, confidence interval.

P^a^ and OR^a^ , acute lung injury vs healthy control; P^b^ and OR^b^ , acute lung injury vs sepsis alone.

* The adjusted P-value and OR in multivariate analyses after adjustment for age, gender, body mass index and history of smoking.
